# Supplementary material for: Fingerprinting conflict: A comparative model with applications to archaeological and historical data
Source: PLoS One. 2025 Feb 21;20(2):e0313895. doi: 10.1371/journal.pone.0313895 (PMC11844905; doi:10.1371/journal.pone.0313895)
Supplement: Attachment — Submitted filename: Comments of the Reviewer.docx [file pone.0313895.s001.docx]

The paper **Fingerprinting** conflict is a valuable study which should be published after major revisions. The following comments are meant to enhance the quality of the paper and inspire the authors to **consider greater complexity of the social phenomenon they studied**. Surely conflicts cannot be lumped into **side 1 vs. side 2**, since **there are more often than not other involved interest groups, sometimes directly, sometimes indirectly involved**. Within each supposed sides there are **individuals and groups with different and conflicting interests during the conflict, both in escalation and de-escalation phases**. Historical examples are numerous.

For example, Second Intermediate Period Egypt (ca. 1650-1550 BCE) in which we have at least three conflicted sides, Thebans, Hyksos and Kushites. The Kushites in one phase of the conflict even involved other African polities as their allies (for example Medjay peoples of Eastern Desert and the Land of Punt from the Horn of Africa). Each side consisted of individuals and groups with different interests. For example, some Egyptians living in Lower Nubian military forts, that previously belonged to Egypt, simply changed sides and became loyal to the king of Kush. Would this be an example of de-escalation, changing sides? At the same time Hyksos offer the Kushites to join them in crushing the Thebans. Is this escalation or de-escalation and for whom? For Kushites this would be escalation since they would be dragged into a conflict of others. For Hyksos this would be de-escalation through victory over the Thebans.

This example serves to illustrate, the danger I see with this paper; it is using an interesting but very simplified model. One of the biggest problems of the paper is **frequent jumping from conflicts between individuals to conflicts between groups**. Furthermore, the paper is in certain parts unnecessarily long, so I suggested to fully remove some parts of the texts.

**Abstract**

The authors write that previous studies of conflicts are rather unnuanced in focus on violence and war. This is simply not true and a simple literature survey demonstrates this. The authors should adjust this rather problematic statement. I will provide examples for nuanced approaches later in these comments.

**Introduction**

44-49 this entire part of the text is not necessary

**Theoretical foundation of conflict research**

52-53 this part of the text is not necessary

54 conflict archaeology exists for several decades already, so it definitely not recent. Please change this.

57-58 references 4, 5, 6 and 7 are all prehistory based and not really up to date. See my comments to **References** below indicating some important studies that were left out. Much work has been done on war and violence in historical archaeologies and the authors should at least include some general references. I made some suggestions in my comments to the **References** which also include historical societies.

65-66 War and peace were not only differently understood in the course of research history, but were differently conceptualized in different cultures. The authors should consult and quote the book of Vanessa Davies, *Peace in Ancient Egypt* (Leiden and Boston: Brill, 2018) where she explains this using ancient Egypt as an example.

67-68 Not only do definitions of war differ, but the conceptualizations of war were different in different societies. To use the words of Judith Butler, war is differently framed in different conflicts and cultures. For examples of frames of war in which conflict is understood as a hunt, a cosmic struggle against demons of the Underworld or even a male domination, see the book of Uroš Matić, *Body and Frames of War in New Kingdom Egypt. Violent Treatment of Enemies and Prisoners* (Wiesbaden: Harrassowitz, 2019).

74-94 this entire section can be removed from the paper.

119-124 what about symbolic violence? For this consult the work of Pierre Bourdieu. For applications in archaeologies of violence see papers in the volume *Archaeologies of Gender and Violence* (Oxford: Oxbow Books, 2017) edited by Uroš Matić and Bo Jensen. These papers and the book *Violence and Gender in Ancient Egypt* (London: Routlede, 2021) by Uroš Matić provide very nuanced approaches to conflicts including a gender perspective.

135-136 This is indeed the main problem of this paper. The model is applied binary, and conflicts are rarely only involving two sides, since these very sides are interpretative constructs. There are so many contexts in which members of one side had different and often conflicting interests.

140-144 this entire part of the text demonstrates an argument based in socio-biology. This has been heavily criticized and should be avoided in a paper which does not deal with socio-biology anyhow. Please delete it.

160 What happens when we have several sides involved?

**The theory section is heavily under referenced! This should be changed.**

**The model**

169 Please do not used the word linguistics, since your case studies dealing with texts did not provide any linguistic analysis. I suppose this is the consequence of the fact that most of the authors are German native speakers. Therefore, please revise English use and find a suitable replacement for linguistics here as it is misleading.

Table 1.

Provide references for the claim that high population density leads to conflicts. Any cross-cultural anthropological studies? If not, be transparent and write that this is an assumption not based in research.

Consider that public sites for democratic processes is a presentist statement since democracy is attested in a very limited number of societies and even in these it has entirely different meaning. For example, Athenian 5^th^ century BCE democracy was much different than modern concept of democracy.

Demolition of settlements. The authors should explain that these are problematic to interpret as only evidence of conflicts. For this see the book of Jesse Millek, *Destruction and Its Impact on Ancient Societies at the End of the Bronze Age* (Columbus: Lockwood Press, 2023).

**Data**

327 all sites are European; the study is clearly Eurocentric and this should be stated.

344 ethnic groups in Bronze Age? How do you justify this? Please consult and quote some seminal studies on issues with applying ethnicity to prehistoric societies, e.g., Sian Jones, *The Archaeology of ethnicity* (London: Routledge, 1997).

410-411 what if this is the evidence for internal conflict?

**Case study Schleswig-Holstein** is too long, it often lacks focus and is missing concluding remarks.

**Case study Poland (Bronze and Iron Age)** is also missing explanations of the observed patterns.

**Case study Crete (Early to Middle Bronze Age)** is too long, especially compared to other case studies. However, this is the first case study which offers some conclusions and explanations. Other case studies should also have these.

700-717 can be deleted, it does not contribute to any provided arguments of the paper

**Case study Rome (Early Republic-Late Empire)** way too lengthy. All of the lengthy sources’ quotations can be removed (1127-1164). The text should focus on the topic and provide summaries.

1214 the statement that the power of Rome was accepted is simply not true and should be deleted. Instead, numerous cases of rebellions against the Roman rule in the provinces should be mention. For example, the uprising in Upper Egypt and Lower Nubia supported by Meroe during the early reign of Augustus. Many more examples can be quoted.

**Case study Hedeby (8^th^ to 11^th^ century)** this is the only case study which acknowledges the possibility of an internal conflict. Other case studies should consider this possibility too and go beyond the binary two side conflict assumption.

**Case study Rus (980-1015 AD)** well written but could be more focused on the topic.

**Case study the Teutonic order (1226-1525 AD)** is considerably shorter than other case studies and written in a different style. The focus is only on 2 parties involved in the conflict. What about other conflicts of these parties with additional parties? Is not the reality more complicated?

**Case study Bohemia (c. 900-1946 AD)** longest chronological range and therefore huge discrepancy compared to other case studies. Did the authors account for these differences in lengths of the periods observed?

**Case study Volga Germans (1763-2000 AD)**

1726 proof read English, the use of the term mother colonies is problematic, it is coming from German.

**Discussion**

1932-1935 These observations need to be explained and not left like this. What does this mean? How do the authors interpret it?

**References**

**Add:**

Debra L. Martin and David F. Frayer, eds. *Violence and Warfare in the Past*. Gordon and Breach Publishers, 1997.

Sarah Ralph, ed. *The Archaeology of Violence: Interdisciplinary Approaches* (Institute for European and Mediterranean Archaeology Proceedings 2, New York: State University of New York Press, 2013).

Andrea Dolfini, Rachel J. Crellin, Christian Horn and Marion Uckelmann, eds. *Prehistoric Warfare and Violence: Quantitative and Qualitative Approaches* (Dordrecht, Heidelberg, London & New York: Springer, 2018).

10. Not a scientific reference! Consider replacement

18. Confirms my assumptions of the influence of sociobiology. Very problematic and not well argued in the paper. Please remove.

**Figures**

Figs. 9 and 10 are not needed.
