# Supplementary material for: Fingerprinting conflict: A comparative model with applications to archaeological and historical data
Source: PLoS One. 2025 Feb 21;20(2):e0313895. doi: 10.1371/journal.pone.0313895 (PMC11844905; doi:10.1371/journal.pone.0313895)
Supplement: Attachment — Submitted filename: Response to Reviewers.docx [file pone.0313895.s002.docx]

Below we indicate (in blue) how we have revised the paper in response to suggestions from the editorial office of the journal and the two reviewers.

**Comments from the editorial office**

Journal requirements:

When submitting your revision, we need you to address these additional requirements.

1. Please ensure that your manuscript meets PLOS ONE's style requirements, including those for file naming. The PLOS ONE style templates can be found at

https://journals.plos.org/plosone/s/file?id=wjVg/PLOSOne_formatting_sample_main_body.pdf and

https://journals.plos.org/plosone/s/file?id=ba62/PLOSOne_formatting_sample_title_authors_affiliations.pdf

Done.

2. Thank you for stating the following financial disclosure:

“This work was supported by the Deutsche Forschungsgemeinschaft (German Research Foundation) under Germany’s Excellence Strategy (grant EXC 2150 390870439).”

Please state what role the funders took in the study.  If the funders had no role, please state: "The funders had no role in study design, data collection and analysis, decision to publish, or preparation of the manuscript."

Done.

If this statement is not correct you must amend it as needed.

Please include this amended Role of Funder statement in your cover letter; we will change the online submission form on your behalf.

Done.

3. We note that Figures 9 and 10 in your submission contain copyrighted images. All PLOS content is published under the Creative Commons Attribution License (CC BY 4.0), which means that the manuscript, images, and Supporting Information files will be freely available online, and any third party is permitted to access, download, copy, distribute, and use these materials in any way, even commercially, with proper attribution. For more information, see our copyright guidelines: http://journals.plos.org/plosone/s/licenses-and-copyright.

We require you to either (1) present written permission from the copyright holder to publish these figures specifically under the CC BY 4.0 license, or (2) remove the figures from your submission:

We have removed the figures, as per a suggestion from Reviewer #2. We agree with the Reviewer that they are not essential.

4. Please review your reference list to ensure that it is complete and correct. If you have cited papers that have been retracted, please include the rationale for doing so in the manuscript text, or remove these references and replace them with relevant current references. Any changes to the reference list should be mentioned in the rebuttal letter that accompanies your revised manuscript. If you need to cite a retracted article, indicate the article’s retracted status in the References list and also include a citation and full reference for the retraction notice.

The reference list has been reviewed. No retracted papers are cited.

**Comments from reviewers**

Reviewer #1: Review
The article consists of several parts that build on each other in a comprehensible way. The first part explains why a new model was developed to describe conflicts in the past. The authors rightly criticize the fact that the discussion has increasingly focused on war and violence in the last years. At least from the perspective of archaeology, I can confirm this. They propose a model in which de-escalation and escalation are examined in order to comparatively investigate (pre-)historical conflict processes. Indicators for de-escalation and escalation are presented as examples and categorized into five levels of a pyramid.
The second part includes ten case studies. They are described on the basis of indicators of de-escalation and escalation, and the archaeological and historical sources are presented. As the archaeological and historical evidence of the individual case studies is very different, the indicators are also heterogeneous. The authors are aware of this and characterize their approach as “generous”: “Nor do we attempt to heavily restrict the assignment of different indicators to different pyramid cells” (lines 1928-29). For each case study, the indicators are listed along an approximate timeline and each indicator is assigned to a specific level of the pyramid.

Thank you for an accurate characterization of our paper.

The case studies are clearly presented. Only the case study ‘trade policies’ in Rome stands out. Here the focus is exclusively on the economic sphere and the economic policies are described in lines 1207-1208 as a potential for conflict. It is thus limited to only one aspect of Roman society in the period of the Early Republic and Late Empire, whereas all other case studies attempt to take into account sources from various areas of (pre-)historical communities and processes.

In the beginning of the text for this case study we added a bit of text stressing that we are being selective, not trying to be comprehensive.

The section with the case studies is purely descriptive but provides an interesting approach to how completely different times and regions can be described using the model of de-escalation and escalation. But is this model also suitable as an analytical tool? The authors address this question in the third and final part of the article. They evaluate the indicators in combination with the levels in the pyramid using several statistical methods. The result of the summary statistics is the ‘fingerprinting’ they also refer to in the title. It is shown in Figure 21 and is easy to understand except for the sometimes difficult to distinguish gray shades.

Again thank you for an accurate characterization. It is not important to distinguish the shades in detail. The underlying data are available if precise numbers are needed.
In connection with 'fingerprinting', the article takes a temporal factor into account, but only to make the levels in the pyramid statistically comparable. The temporal dimension is my main criticism of this manuscript, as it is not discussed critically enough. The individual case studies show various processes or events that are classified as de-escalating or escalating. However, in most cases it is not clear whether and to what extent de-escalating and escalating indicators are connected in time or even represent a reaction within particular processes. In the case study of the Volga-Germans, for example, indicators of escalation can be found up to 1875, while there are no indicators of de-escalation in the same period. Does this mean that no de-escalation measures were taken until well into the 19th century? Generally, the question arises how and to which extent certain indicators are connected in time and may have led to an increase/decrease in escalation and de-escalation. Direct correlations can certainly only ever be assumed - especially in prehistoric times.

Establishing causation in history is a can of worms. But actually we currently keep a distance to this problem, although this was perhaps not made clear enough. Now we added the following text to the end of the section called ‘The model’: “While it is fair to assume that for a given conflict there is some degree of interrelatedness of actions corresponding to the various indicators observed, i.e. a network of causality—perhaps with some events that are more central than others—it not a requirement of the model that two indicators are in fact related. For instance, in a given conflict some act of threat (escalation Level 3) may be identified as well as some act of violence (Level 4), and both would then be registered in the descriptive model. But the description is a static one, so there is no necessity of an assumption of direct causation between the act of threat and the act of violence. In a further development of the model a dynamic perspective may conceivably be introduced, allowing for the identification of diachronic patterns of conflict developments. This is a matter for future work, however.”

But I think this is an important point that the authors should discuss critically because such considerations are particularly interesting for the interpretation of the following statistical analyses. Fig. 22 shows the result of the analysis of the weighted means, but here too the indicators for de-escalation and escalation were evaluated in the respective case studies, always taking the entire period into account.

An analysis such as the one in Fig. 22 is consistent with our static approach, which is now made more explicit in the text added in response to the previous comment. We are aware that the approach is reductionist inasmuch as the diachronic perspective is collapsed. Since we already state once that a dynamization of the model is (potentially) a matter of future research we won’t repeat that in the text accompanying Fig. 22, but we are aware of the limitations.
A revision of Figure 23 ‘Spearman correlation’ is advisable, as ‘Schleswig-Holstein’ is missing.

Schleswig-Holstein was missing because of an absence of a correlation in the de-escalation dimension, which was interpreted by the R function as NA (“not applicable”). This was replaced by a zero, now allowing for the inclusion of Schleswig-Holstein. Thank you for this very acute observation.

Secondly, the four ‘ideal types’ in the same figure should be added graphically to make it easier to understand.

We have now amended the figure with annotations attached to the four corners and more verbose axis labels to make it easier to understand.

The following conclusion is derived from this analysis: “different combinations of gradients in escalation or de-escalation exist, but we do not observe a combination of strongly represented lower escalation levels and strongly represented higher levels of escalation, suggesting the hypothesis that the safest way to avoid high levels of escalation like outbreaks of violence may be to focus on basic (lower) levels of de-escalation like cooperation and regulation” (lines 1996-1999). I consider this to be problematic, as the temporal and causal relationships for the case studies have not been discussed in detail or are often not clear from the archaeological and historical sources.
This is of course a problem that is inherent to the sources and cannot be solved. But I would therefore advise the authors to comment in detail on the temporality of the indicators. The aim stated in the abstract is 'to identify typical patterns in conflict situations'. They are still formulated very abstractly in this manuscript, however convincing the 'finger printing' in the case studies may be.

We now appended the following remarks to the end of the Discussion section, again raising issues also discussed in new text at the end of the section ‘The model’.

“We do want to add, though, that these results stem from a model which represents a strong simplification of reality inasmuch as the diachronic dimension has been collapsed and causal relationships, although broadly assumed to exist, have not been established or explored. Further research applying a dynamized version of the E–D Pyramid model as well as more data would be necessary for confirmation of the findings.”

Some suggestions for corrections:
Keywords: delete ‘war’ and ‘peace’, replace with ‚escalation‘ and ‚de-escalation’

Done.
line 49: ‘Finally we offer a Conclusion’, please contribute a little bit more to this sentence.

This and surrounding text was deleted entirely, following the recommendation of Reviewer #2.
line 238: ‘but it also be imply …’ Delete ‘be’

Done.
line 268: two times ‘representing’

The second occurrence now replaced by “implying”.
Lanzenspitzen, better ‘spearheads’ (please check)

We couldn’t find the word ‘Lanzenspitzen’ in our text, but in line 406 we used ‘lance points’. This has been changed to ‘lance heads’.
line 426: ‘burial constructions’, not ‘custom’ when you are writing about burial mounds

Done.
Case study: Poland: in the text you discuss cooking pits as gathering places, but you do not show them as indicators of de-escalation in Fig. 7?

As was/is mentioned in the text (line 533 of the original submission) there is a lack of studies of the phenomenon for Poland, so we hesitated to make cooking pits an element in the formal analysis. We also now downplayed them by removing ‘cooking pits’ from the title of the case study.
line 604: ‘the subsistence strategy changed’ instead of ‘a changed subsistence strategy emerged’

Done.
Case study: Hedeby: Please use the same terms in the text and in Fig. 15. In Fig. 15 two times ‘signal fires’. The first time it is given as an indicator of escalation, but without a symbol

The list of indicators in the text and the designations as they appear in Fig. 15 are now the same and ‘signal fires’ appears only once. A lack of symbols (=timeline) occurs when a given indicator cannot be dated. This was/is mentioned in the caption to the figure.
Case study: Rus’: Fig. 16 – Why are the events of ‘Byzantine military services’ not given in one line according to the time line?

This and some other indicators that appear discontinuously in the record are put on separate lines partly for technical reasons, partly because circumstances might be different.
line 1482-38 and Fig. 16: familiar missionary language: should it be assigned only descalating, according to the text?

Fig. 16 shows a grey color, reflecting the fact that both de-escalation level 2 and escalation level 2 received the highest confidence (a ‘1’). As correctly observed, the thinking behind this scoring is not reflected in the text. To the statement “The linguistic proximity between early East Slavic and Old Church Slavonic would have had a positive effect on the willingness to accept Christianity” we therefore now add “but language standardization usually also comes with a cost of disputes over linguistic norms.”

Thank you for some very insightful comments!

Reviewer #2

The paper **Fingerprinting** conflict is a valuable study which should be published after major revisions. The following comments are meant to enhance the quality of the paper and inspire the authors to **consider greater complexity of the social phenomenon they studied**. Surely conflicts cannot be lumped into **side 1 vs. side 2**, since **there are more often than not other involved interest groups, sometimes directly, sometimes indirectly involved**. Within each supposed sides there are **individuals and groups with different and conflicting interests during the conflict, both in escalation and de-escalation phases**. Historical examples are numerous.

For example, Second Intermediate Period Egypt (ca. 1650-1550 BCE) in which we have at least three conflicted sides, Thebans, Hyksos and Kushites. The Kushites in one phase of the conflict even involved other African polities as their allies (for example Medjay peoples of Eastern Desert and the Land of Punt from the Horn of Africa). Each side consisted of individuals and groups with different interests. For example, some Egyptians living in Lower Nubian military forts, that previously belonged to Egypt, simply changed sides and became loyal to the king of Kush. Would this be an example of de-escalation, changing sides? At the same time Hyksos offer the Kushites to join them in crushing the Thebans. Is this escalation or de-escalation and for whom? For Kushites this would be escalation since they would be dragged into a conflict of others. For Hyksos this would be de-escalation through victory over the Thebans.

This is really interesting. We would have loved to include a Egyptian case study if your expertise had been available to us. Thank you for bringing a concrete example to the table. We completely agree that modelling should take into account the possibility of multiple partners as well as allow for multiple perspectives. In a paragraph added a couple of pages from the end of the section called ‘The model’ we now discuss these issues as follows (reflecting your example but in more abstract terms since we lack expertise to properly present the concrete case that you mention): “… there is nothing that hinders an application of the model to more complex scenarios involving multiple partners. Interpretations then also become more complicated and perhaps more prone to ambiguities. For instance, given a conflict between A and B, should we interpret the event of an alliance between A and a third partner, C, as representing escalation or de-escalation? The decision would depend on whether the event is more likely to aggravate the situation or not, which is of course a matter of interpretation. The model also allows for flexibility with respect to the point of view. In the case studies of this paper we try take a neutral point of view, looking at conflicts from the ‘outside’. But a conflict might also be modelled from the ‘inside’, taking the view of a particular partner. Let us again refer to the fictional example with the newly formed alliance of A and C. Even in the case where this is expected to bring more stability and is therefore deemed de-escalatory from the point of view of the entire situation, it may still be seen as escalatory from the point of view of C in case this partner was not in any way affected by the conflict prior to the alliance.”

This example serves to illustrate, the danger I see with this paper; it is using an interesting but very simplified model.

As we try to indicate in the added text just quoted the model may be simple, but it is at the same time flexible enough to accommodate complex realities.

One of the biggest problems of the paper is **frequent jumping from conflicts between individuals to conflicts between groups**.

We do sometimes refer to acts of individuals, such as Vladimir in the Rus’ case study. But, as was stated already in the original submission (lines 148-150), conflicts may involve both groups and individuals: “A conflict situation exists when at least two parties (groups or individuals) have potentially incompatible wishes or interests.” Now we furthermore add the following statement towards the end of the section called ‘The model’: “Most cases exclusively concern whole groups (societies), but our model is impervious to whether agents are constituted by individuals or groups. That said, we regard history as driven more by groups than by individuals.”

Furthermore, the paper is in certain parts unnecessarily long, so I suggested to fully remove some parts of the texts.

We have followed the concrete recommendations below regarding text to be deleted and additionally found some more text here and there that could be dispensed with.

**Abstract**

The authors write that previous studies of conflicts are rather unnuanced in focus on violence and war. This is simply not true and a simple literature survey demonstrates this. The authors should adjust this rather problematic statement. I will provide examples for nuanced approaches later in these comments.

We changed this to “Studies of conflicts in these fields have often focused on violence and war”, which is hardly controversial.

**Introduction**

44-49 this entire part of the text is not necessary

This was radically reduced to just a single sentence.

**Theoretical foundation of conflict research**

52-53 this part of the text is not necessary

Now deleted.

54 conflict archaeology exists for several decades already, so it definitely not recent. Please change this.

We did write “relatively recently”. But we realize that we should be more specific, so now we write “Although conflict archaeology has emerged as a sub-discipline in its own right only during the last three decades...”. We added a reference to a paper by Vandkilde from one of the edited volumes recommended (Ralph 2013).

57-58 references 4, 5, 6 and 7 are all prehistory based and not really up to date. See my comments to **References** below indicating some important studies that were left out. Much work has been done on war and violence in historical archaeologies and the authors should at least include some general references. I made some suggestions in my comments to the **References** which also include historical societies.

We added references to the three books suggested (Martin and Frayer 1997, Ralph 2013, Dolfini et al. 2018). Great suggestions! All of them count among works that have a focus on the violent side of conflicts.

65-66 War and peace were not only differently understood in the course of research history, but were differently conceptualized in different cultures. The authors should consult and quote the book of Vanessa Davies, *Peace in Ancient Egypt* (Leiden and Boston: Brill, 2018) where she explains this using ancient Egypt as an example.

67-68 Not only do definitions of war differ, but the conceptualizations of war were different in different societies. To use the words of Judith Butler, war is differently framed in different conflicts and cultures. For examples of frames of war in which conflict is understood as a hunt, a cosmic struggle against demons of the Underworld or even a male domination, see the book of Uroš Matić, *Body and Frames of War in New Kingdom Egypt. Violent Treatment of Enemies and Prisoners* (Wiesbaden: Harrassowitz, 2019).

In response to these comments on 65-66 and 67-68 we updated the text so as to include the suggested examples, as follows (where the last two references are to Davies 2018 and Matić 2019, respectively): “The concepts of ‘war’ and ‘peace’ represent significant theoretical challenges, as they have been understood and distinguished in many different ways during the history of research [Refs]. Moreover, these concepts may be conceptualized in different ways across cultures. For instance, the closest translational equivalent of ‘peace’ among the Ancient Egyptians is *hetep*, a concept which did not necessarily exclude violence, but rather referred to action in accord with the proper order of life on Earth and in the cosmos [Ref], and a frequent conceptualization of war is a hunting simile [Ref].”

74-94 this entire section can be removed from the paper.

Done.

119-124 what about symbolic violence? For this consult the work of Pierre Bourdieu. For applications in archaeologies of violence see papers in the volume *Archaeologies of Gender and Violence* (Oxford: Oxbow Books, 2017) edited by Uroš Matić and Bo Jensen. These papers and the book *Violence and Gender in Ancient Egypt* (London: Routlede, 2021) by Uroš Matić provide very nuanced approaches to conflicts including a gender perspective.

We revised 119-124 now also mentioning symbolic violence. Given the Editor’s recommendation to avoid new references we kept additions minimalistically to a single reference to Bourdieu & Wacquant 2004. The revised text is as follows: “Where we do specify violence terminologically as a component of our model we reserve the term for physical violence. Nevertheless, there are various non-physical forms of violence which also represent escalative behavior. As first defined by Galtung [26], structural violence manifests itself when a social structure or institution inflicts harm upon individuals by obstructing their ability to fulfil fundamental needs, and symbolic violence, as described by Bourdieu [27], covers forms of repression that that operate at a communicative level and are so subtle that they may not even be recognized by their victims.”

135-136 This is indeed the main problem of this paper. The model is applied binary, and conflicts are rarely only involving two sides, since these very sides are interpretative constructs. There are so many contexts in which members of one side had different and often conflicting interests.

As mentioned in our first reply above we now state more explicitly that out model is not binary and does not have to be applied in such a way.

140-144 this entire part of the text demonstrates an argument based in socio-biology. This has been heavily criticized and should be avoided in a paper which does not deal with socio-biology anyhow. Please delete it.

We deleted most of 140-144, but the discussion still revolves around how conflicts may escalate beyond the threshold where everyone loses out. We don’t think that it entails a socio-biological stance to assume that conflicts involve rational agents whose actions are mainly guided by what’s advantageous for them.

160 What happens when we have several sides involved?

Instead of “a third party” we now write “other parties”.

**The theory section is heavily under referenced! This should be changed.**

As indicated above more references have now been added.

**The model**

169 Please do not used the word linguistics, since your case studies dealing with texts did not provide any linguistic analysis. I suppose this is the consequence of the fact that most of the authors are German native speakers. Therefore, please revise English use and find a suitable replacement for linguistics here as it is misleading.

We simply deleted “linguistics”.

Table 1.

Provide references for the claim that high population density leads to conflicts. Any cross-cultural anthropological studies? If not, be transparent and write that this is an assumption not based in research.

We added a brief statement below the table indicating that increasing population density does not necessarily cause escalation, but that it may add to the probability of disputes, citing Rappaport (1966) on the Tsembaga Maring (New Guinea), and the cross-cultural studies of Alberti (2014) and Ember & Ember (2014).

Consider that public sites for democratic processes is a presentist statement since democracy is attested in a very limited number of societies and even in these it has entirely different meaning. For example, Athenian 5^th^ century BCE democracy was much different than modern concept of democracy.

In the table we changed “Public sites for democratic processes” to “Public sites for negotiation processes”.

Demolition of settlements. The authors should explain that these are problematic to interpret as only evidence of conflicts. For this see the book of Jesse Millek, *Destruction and Its Impact on Ancient Societies at the End of the Bronze Age* (Columbus: Lockwood Press, 2023).

After the table we have add a cautionary remark to this effect, citing Millek (2023).

**Data**

327 all sites are European; the study is clearly Eurocentric and this should be stated.

We now write “…the expertise available of course ultimately guided the temporal and geographical selection, the latter of necessity being Eurocentric.”

344 ethnic groups in Bronze Age? How do you justify this? Please consult and quote some seminal studies on issues with applying ethnicity to prehistoric societies, e.g., Sian Jones, *The Archaeology of ethnicity* (London: Routledge, 1997).

This is simply a misunderstanding. We didn’t wish to imply anything about ethnic groups in the Bronze Age. We just wrote that the titles of our case studies could refer to areas or ethnic groups. This was simply intended to also cover the section on Volga Germans whose title refers to an ethnic group (the Bronze age studies all have geographical names in their titles). In order to avoid the misunderstanding we now say that the titles can refer to areas or topics.

410-411 what if this is the evidence for internal conflict?

No attempt is made to distinguish ‘internal’ vs. ‘external’ conflict and the model does not require any such distinction. So the best answer is probably that it is not really an issue.

**Case study**

**Schleswig-Holstein** is too long, it often lacks focus and is missing concluding remarks.

We deleted more than 350 words, mainly from the beginning of the case study, and added a short paragraph summarizing our observations.

**Case study Poland (Bronze and Iron Age)** is also missing explanations of the observed patterns.

Here we added a couple of sentences to the last paragraph, improving on the summary of the observed patterns.

**Case study Crete (Early to Middle Bronze Age)** is too long, especially compared to other case studies. However, this is the first case study which offers some conclusions and explanations. Other case studies should also have these.

This was shortened by about 400 words.

700-717 can be deleted, it does not contribute to any provided arguments of the paper

Done.

**Case study Rome (Early Republic-Late Empire)** way too lengthy. All of the lengthy sources’ quotations can be removed (1127-1164). The text should focus on the topic and provide summaries.

All the lengthy quotes have been removed and text is now around 350 words shorter.

1214 the statement that the power of Rome was accepted is simply not true and should be deleted. Instead, numerous cases of rebellions against the Roman rule in the provinces should be mention. For example, the uprising in Upper Egypt and Lower Nubia supported by Meroe during the early reign of Augustus. Many more examples can be quoted.

We changed “accepted” to “maintained” and provide many (seven to be exact) examples of rebellions.

**Case study Hedeby (8^th^ to 11^th^ century)** this is the only case study which acknowledges the possibility of an internal conflict. Other case studies should consider this possibility too and go beyond the binary two side conflict assumption.

The recommendation for revision of other case studies is rather broad and would lead to rewriting most of them, something which is not feasible. Moreover, any treatment of a conflict will be reductionist to some extent, since, in a connected world, no conflict is contained in time and space even if the description has to come to a halt at some point.

**Case study Rus (980-1015 AD)** well written but could be more focused on the topic.

No action taken.

**Case study the Teutonic order (1226-1525 AD)** is considerably shorter than other case studies and written in a different style. The focus is only on 2 parties involved in the conflict. What about other conflicts of these parties with additional parties? Is not the reality more complicated?

Text has been added to indicate that there are, indeed, other parties in the periphery of the conflict, making it clearer that reality is in fact more complicated.

**Case study Bohemia (c. 900-1946 AD)** longest chronological range and therefore huge discrepancy compared to other case studies. Did the authors account for these differences in lengths of the periods observed?

The chronological ranges can be read off from the timeline figures or be directly be extracted from the supplementary information. They range from 237 years (Volga Germans) to 2550 (Sambia), with the 1099 year time span for Bohemia being close to the average, which is 996 years. So, yes, there are great differences, even if this particular case study is not out of the ordinary. Towards the end of the section ‘The model’ we added the following: “We believe that similar dynamics are expected to play out in short-term conflicts and longer-term ones, which is why we decided not to introduce distinctions in the temporal dimension. But we are aware that more research would be needed to fully justify this decision.” So we assume that we are not comparing apples and oranges, but we can’t presently justify this assumptions very well. Still, we now at least make the assumption more explicit.

**Case study Volga Germans (1763-2000 AD)**

1726 proof read English, the use of the term mother colonies is problematic, it is coming from German.

The term “mother colonies” was changed to “colonies”, and a few other small, stylistic edits were made as well.

**Discussion**

1932-1935 These observations need to be explained and not left like this. What does this mean? How do the authors interpret it?

We added some down-to-earth remarks since some deeper, more unified explanation does not seem necessary: “Working our way up from the bottom of the pyramid we can easily account for this pattern applying common sense (we will use e1-e5 to refer to escalation levels and d1-d4 to refer to de-escalation levels). Conflict potential (e1) and cooperation (d1) are basic ingredients in social interaction that are omnipresent and expected to often be long-lived. Interfering demands (e2) and regulation (d2) are more subject to fluctuations, but it comes as no surprise that they may also be long-lived. Threats (e3) and conciliation/negotiation (d3) inherently have shorter lives, as they will tend to give way to other levels. For instance, threats may escalate into violence (e4) and negotiation may lead to regulations (d2). Physical violence (e4) is inherently short-lived, probably because it will relatively quickly lead to a reaction, implying a change of levels, and the same would be true of capitulation. Extinction (e5) has to be relatively quick to be efficiently accomplished.”

**References**

**Add:**

Debra L. Martin and David F. Frayer, eds. *Violence and Warfare in the Past*. Gordon and Breach Publishers, 1997.

Sarah Ralph, ed. *The Archaeology of Violence: Interdisciplinary Approaches* (Institute for European and Mediterranean Archaeology Proceedings 2, New York: State University of New York Press, 2013).

Andrea Dolfini, Rachel J. Crellin, Christian Horn and Marion Uckelmann, eds. *Prehistoric Warfare and Violence: Quantitative and Qualitative Approaches* (Dordrecht, Heidelberg, London & New York: Springer, 2018).

Done (all three added).

10. Not a scientific reference! Consider replacement

This reference to an online resource was now replaced with Werkner (2020), which includes an overview of war definitions in modern conflict research, and Peter-Röcher (2007), which provides a more in-depth survey across all relevant disciplines (and was already in the References).

18. Confirms my assumptions of the influence of sociobiology. Very problematic and not well argued in the paper. Please remove.

This has been removed.

**Figures**

Figs. 9 and 10 are not needed.

They have been removed.

Thank you for the many good suggestions!
